# Supplementary material for: HMOX1 genetic polymorphisms and outcomes in infectious disease: A systematic review
Source: PLoS One. 2022 May 12;17(5):e0267399. doi: 10.1371/journal.pone.0267399 (PMC9098073; doi:10.1371/journal.pone.0267399)
Supplement: S1 File — (DOCX) [file pone.0267399.s001.docx]

Search Strategy:

("heme oxygenase" or HMOX1 or "haem oxygenase" or "heme-oxygenase" or "haem-oxygenase" or "HO-1" or “heme-oxygenase-1” ) AND

(repeat or polymorphism or variant or mutation or SNP or microsatellite or GT or genotype )
