# Supplementary material for: HMOX1 genetic polymorphisms and outcomes in infectious disease: A systematic review
Source: PLoS One. 2022 May 12;17(5):e0267399. doi: 10.1371/journal.pone.0267399 (PMC9098073; doi:10.1371/journal.pone.0267399)
Supplement: Attachment — Submitted filename: Response to reviewers.docx [file pone.0267399.s005.docx]

Dear Editors,

PONE-D-21-27041 : HMOX1 genetic polymorphisms and outcomes in infectious disease: a systematic review

Many thanks for considering this article. Please find our response to reviewer comments below. In addition, we would like to state that the funders had no role in study design, data collection and analysis, decision to publish, or preparation of the manuscript, and we have edited the manuscript accordingly.

Please find comments to reviewers below:

Many thanks

Fergus Hamilton, on behalf of the authors.

Reviewer 1

*Reviewer #1: This paper is a systematic review of HMOX1 polymorphisms associated to infectious diseases. The topic is interesting and it was well addressed.*

Many thanks for this kind comment.

*Strengths
The authors registered their paper in PROSPERO, use HuGE guidelines for genetic studies and PRISMA strategy for some findings. The findings are depicted in logical steps.
The use of kernels for probability density was very illustrative for comprehension of the distribution of repeats in and outside Africa.*

Many thanks for these comments.

*Weakness.*
*Just some minor suggestions:
Table 1 is an important source to understand differences between studies. Can the authors add in Definitions of cohorts the mean age (SD) to compare across studies?*

Many thanks for this comment. We have now added this data, where it is available. For some studies, this was either not available or not in a mean (SD) format.

*Did the studies describe sex differences regarding the frequency of outcomes? For example, HIV patients’ trend is to have a high frequency of males. Another modifier of effect is race or ethnic groups that the authors discussed extensively in the different sections.*

The studies do not report a sex difference in the outcome. We have added some commentary in the discussion around this. As you say, there is a propensity for certain infections to have a sex predominance, but no study reported an interaction between HMOX1 repeat length and sex.

*Figure 1 has an empty space (typo) for the Reports of included studies (bottom line).*

Many thanks for this. We have edited the PRISMA diagram as we had found another error and redrawn this figure.
